# Length of hospitalization is associated with selected biomarkers (albumin and lymphocytes) and with co-morbidities: study on 4000 patients

**DOI:** 10.1186/s40364-017-0091-x

**Published:** 2017-03-21

**Authors:** Antonio E. Pontiroli, Lara Loreggian, Marco P. L. Rovati, Elena De Patto, Laura Folini, Federico Raveglia, Matilde De Simone, Alessandro Baisi, Ugo Cioffi

**Affiliations:** 1 2nd Division of Medicine, Ospedale San Paolo and University of Milan, Milan, Italy; 2 2nd Division of Surgery, Ospedale San Paolo and University of Milan, Milan, Italy; 3 Division of Thoracic Surgery, Ospedale San Paolo and University of Milan, Milan, Italy; 4 Department of Surgery, University of Milan, Milan, Italy

**Keywords:** Albumin, Lymphocytes, Length of stay, Co-morbidities, Screening method

## Abstract

**Background:**

Low albumin levels and low lymphocyte counts are intra hospital conditions that exert a negative influence on prognosis, healing and length of hospitalization. The study aimed to analyze the correlation between low blood levels of albumin, low lymphocytes, and length of stay. The secondary aim was to identify other co-morbidities associated with prolonged hospital stay.

**Methods:**

Retrospective pilot study was conducted by analyzing anamnestic and biochemical data, related to 4038 patients admitted to ten wards of Hospital San Paolo (Milan), collected from July 1^st^ 2012 to December 31^st^ 2012. A statistical analysis was carried out using the Correlation method, Multivariate Analysis and Regression. Lymphocyte count and co-morbidities were evaluated in the whole cohort, albumin levels in 1437 patients.

**Results:**

In the whole sample, low albumin levels and low lymphocyte counts were directly correlated to longer hospitalizations. The stratification of the results by department and diagnosis suggests that there is a higher correlation in certain subpopulations, and albumin shows a greater correlation with length of stay than lymphocytes. Also advanced age, high platelets, type of diagnosis, male gender and emergency admission led to longer hospitalizations.

**Conclusions:**

A routine check of albumin, lymphocytes and a spectrum of significant variables can provide precious information which can eventually lead to a shorter hospital stay. Knowledge of the general health status of a patient and the possibility to estimate his/her length of hospital stay are essential information for Clinical Governance, and for the improvement of internal services of hospitals on a large scale.

## Bullet points

1. Low levels of albumin and lymphocytes are highly correlated with prolonged hospitalizations.
2. Platelets, advanced age, number of co-morbidities, diagnosis, gender, urgent admission are also associated with prolonged hospitalizations
3. This routine screening will improve the hospitals internal healthcare along with helping to better allocate hospital resources in terms of budget and personnel; even for a clinical audit.

## Introduction

### Background

According to recent studies low albumin levels and low lymphocyte counts are the frequent in hospitalized patients, representing negative conditions with a negative impact on prognosis and length of stay (LOS) [1, 2]. Actually, albumin and lymphocyte count are biochemical parameters successfully used in common clinical practices for highlighting and monitoring certain specific diseases [3]. Because of their importance in human biology and the variety of biochemical processes in which they take part, these markers are indices of general organic damage, unfavourable prognosis and malfunction; even if their role in determining nutritional status and length of stay is still debated [2, 4–14]. The ultimate goal of this study is to raise awareness among physicians about the health status of hospitalized patients, based on lymphocyte count and albumin levels at admission.

### Objectives

The main objective is to verify the correlation between serum albumin levels, lymphocyte counts and length of stay in a vast hospital population, together with various blood parameters and patient history. We would like to create an innovative, economical and standardized screening method, based on albumin, lymphocyte count and an analysis of co-morbidities, in order to assess the organic status of patients and predict the length of recovery at admission. These informations would give the opportunity to improve the organization of hospitals internal services, giving patients better treatments and shortening hospitalizations and reducing social costs associated with long waiting times and incorrect diagnosis [15, 16].

## Methods

### Study design, setting and participants

This is a retrospective cohort study, which took place in Hospital San Paolo of Milan after the study protocol was approved by the local Ethics Committee. The study included all the patients in the following wards: Surgery (1^st^ and 2^nd^ ward), Gynecology, Obstetrics, Infectious Diseases, Medicine (1^st^, 2^nd^, and 3^rd^ ward), Orthopedics, and Urology. The observation period was July 1^st^ 2012 - December 31^st^ 2012. Day Hospital patients were excluded from the study (since the study tested how low levels of albumin and lymphocytes influenced the length of stay). Data collection, through electronic charts, included recording of anamnestic data such as gender, age, date of admission and discharge, type of diagnosis, concomitant diseases (identified by the specific International Classification Code of Diseases) and biochemical indices like complete blood count with lymphocytes count and albumin measured during the first 24–48 h. Starting from electronic charts, we created an Excel database containing all informations registered for all patients, divided according to the ward of admission. Raw data was critically examined using the method of descriptive statistical analysis, and the results were stratified by type of admission (elective or urgent), by ward, and diagnosis.

**Fig. 1:**
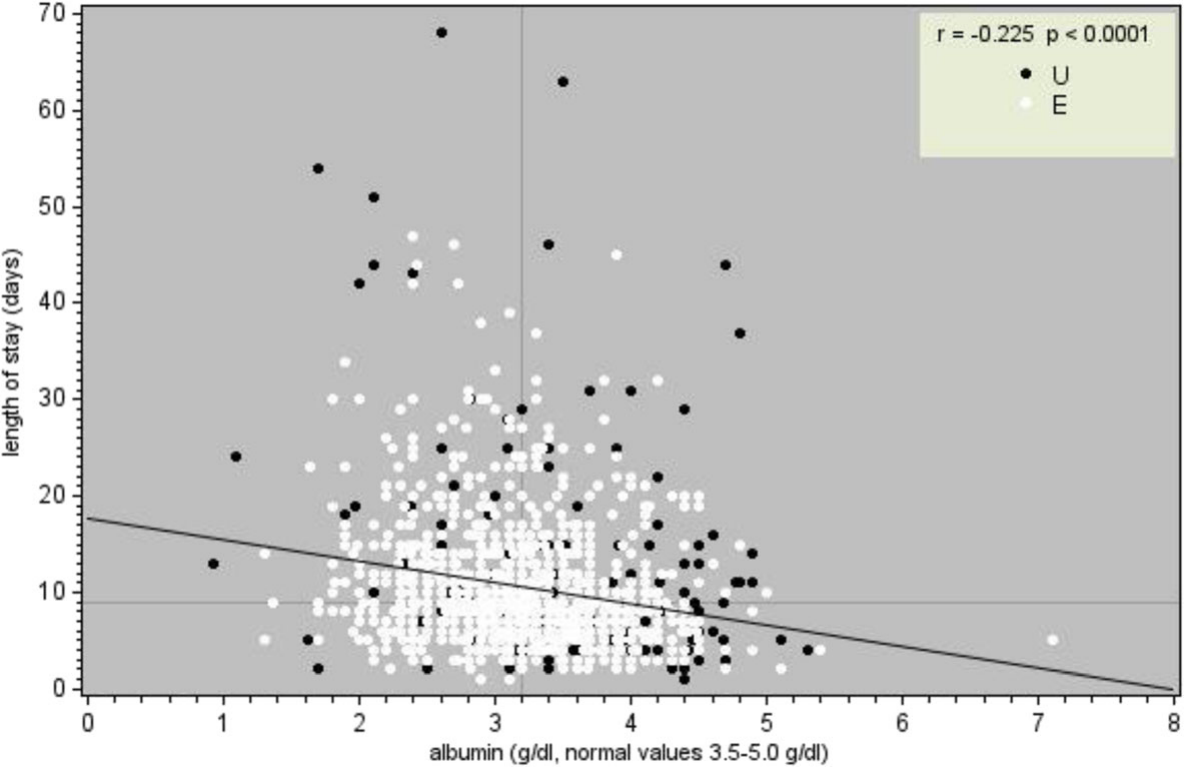
Relationship between length of stay (days) and serum albumin levels (g/dl). Legend. *White circles* indicate patients with emergency admission; *black circles* indicate patients with routine admission

**Fig. 2:**
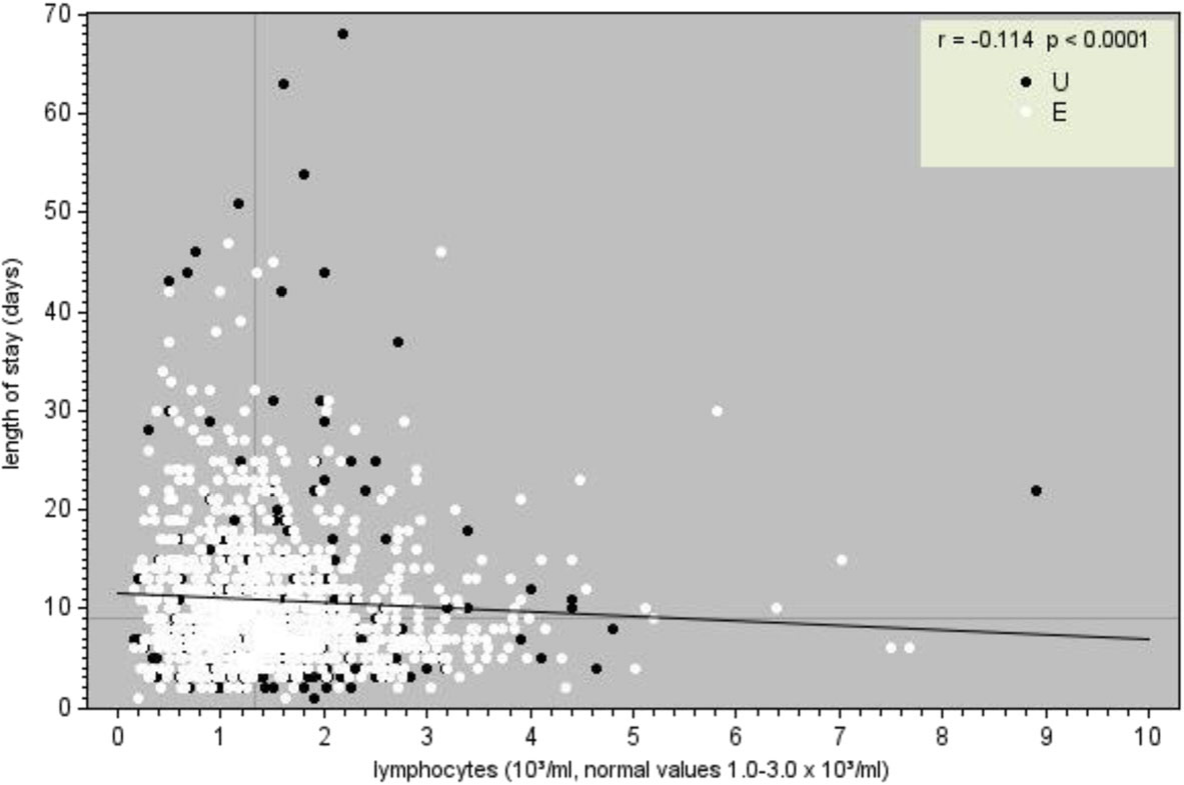
Relationship between length of stay (days) and lymphocite count (10^3^/ml). 1.0–3.0). Legend. *White circles* indicate patients with emergency admission; *black circles* indicate patients with routine admission

In this study the ranges of albumin and lymphocytes considered normal were 3.5-5.0 g/dL and 1.0-3.0 x 10^3^/ml respectively, as described in Figs. 1 and 2. Consequently, levels of albumin and lymphocytes under 3.5 g/dl and 1.0 x 10^3^/ml were considered low.

### Statistical analysis

The SAS 9.3 software and its related modules (SAS MACRO and SAS GRAPHS) were used for data cleansing, statistical models, descriptive statistics and graphs. JMP 11 was also used to enhance the models and the graphs. Using the Correlation method we studied the relationship between albumin, lymphocytes, and length of stay, verifying its extensibility in the various wards, then divided the patients by diagnosis and lastly studying the entire hospital population. To obtain a good statistical analysis we include the most popular wards. The sample was then divided into eight main “classes of diagnosis”, based on the main types of diseases observed in our hospital population: Malignant tumors, Benign tumors, Inflammatory diseases, Infections, Bleeding conditions, Fractures, Pregnancy/Childbirth and Other. Even in this case statistical analysis was performed in order to examine type and intensity of the relationship between the hospital stay and low albumin and lymphocytes levels. At a later stage we used Regression analysis to highlight which other factors could influence length of stay, using Generalized Linear Models method (GLM). Finally, we studied the behavior of the variable “days of hospitalization” as a function of the number of co-morbidities. Correlation was evaluated using Pearson’s parametric coefficient while bivariate distribution was graphically represented using scatter plots.

For a more detailed and complete analysis we evaluate if there is a difference between two particular subgroups of the test sample: patients in which the albumin was measured and patients who do not present the value. The methodologies used to study the two groups were the Discriminant function analysis and the Multivariate stepwise method.

## Results

### Analysis on the entire sample

During the period of study 4038 people matched the inclusion criteria, thus distributed in hospital wards: Surgery 1^st^ (*n* = 411), Surgery 2^nd^ (*n* = 343), Gynecology (*n* = 149), Obstetrics (*n* = 1184), Infectious Diseases (*n* = 206), Medicine 1^st^ (*n* = 550), Medicine 2^nd^ (*n* = 345), Medicine 3^rd^ (*n* = 291), Orthopedics (*n* = 278), Urology (*n* = 281). Table 1 shows that some wards had a high rate of cases of albumin evaluation, here described in descending order: Medicine 1^st^, Infectious Diseases, Medicine 3^rd^, Medicine 2^nd^, and Surgery 2^nd^. Instead Orthopedics and Surgery 1^st^, followed by Urology, Gynecology and Obstetrics, had a very low rate of cases with evaluation of albumin. The measurement of albumin in the early hours of admission occurred only in 36% (1437 on 4038, i.e. 1 out of 3) of the total cases, but lymphocyte count appeared to be a routine test, being measured in the totality of the cases (*n* = 4038). As shown in Table 2 the group consists of 1477 men and 2561 women, with age ranging from 8 to 105 years. Within this hospital population, the average age was 60.27 years (SD 22.90) and the median age was 60.81 years (1^st^ interquartile = 38.64; 3^rd^ interquartile = 81.65). Table 3 shows the proportion of patients whose albumin and lymphocytes levels were within the normal range at admission. There was a significant relationship between low albumin levels, low lymphocyte counts and length of stay in the whole sample (Figs. 1 and 2). From a statistical point of view, decreased albumin and decreased lymphocytes significantly correlate with an increase in hospital stay. We also observed the existence of a significant correlation between days of hospitalization and the number of co-morbidities (*r* = 0.4207; *p* < 0.0001) taking into account other ten variables (Age, Red Blood Cell, Hemoglobin, Hematocrit, Mean Cell Volume, Mean Corpuscular Hemoglobin, Mean Corpuscular Hemoglobin Concentration, Platelets, White Blood Cell and Neutrophils) see (Table 4).

**Table 1:** Evaluation of lymphocytes and albumin performed within 24–48 h of admission

Ward	Patients	Albumin evaluated	% Evaluation
Surgery 1^st^	411	101	25%
Surgery 2^nd^	343	160	47%
Gynecology	149	7	5%
Obstetrics	1184	35	3%
Infectious Diseases	206	150	73%
Medicine 1^st^	550	432	79%
Medicine 2^nd^	345	237	69%
Medicine 3^rd^	291	206	71%
Orthopedics	278	71	26%
Urology	281	38	14%
Total	4038	1437	36%

Lymphocytes were evaluated in all patients (100%)

**Table 2:** Main patient’s characteristics

Ward	Total patients	Nr. Male	Nr. Female	Minimum Age	Maximum Age	Mean Age	Median Age
Surgery 1^st^	411	235	176	18	102	63.95 ± 19.67	66.90
Surgery 2^nd^	343	198	145	8	102	66.58 ± 17.80	71.59
Gynecology	149	0	149	19	93	49.72 ± 16.58	45.72
Obstetrics	1184	0	1184	15	94	36.17 ± 6.93	36.35
Infectious diseases	206	130	76	20	90	55.57 ± 17.04	54.25
Medicine 1^st^	550	262	288	22	105	80.29 ± 14.61	84.24
Medicine 2nd	345	178	167	22	104	79.59 ± 14.92	83.10
Medicine 3rd	291	139	152	21	105	79.57 ± 15.91	83.83
Orthopedics	278	125	153	8	102	68.01 ± 21.88	73.03
Urology	281	210	71	22	96	67.74 ± 16.13	70.98
Total	4038	1477	2561	8	105	60.27 ± 22.90	60.81

**Table 3:** Frequency of albumin and lymphocytes cases within normal range of laboratory

Ward		Albumin			Lymphocytes	
	Total patients	Within normal range	% Normal range	Total patients	Within normal range	% Normal range
Surgery 1^st^	101	53	52%	411	314	76%
Surgery 2^nd^	160	78	49%	343	230	67%
Gynecology	7	0	0%	149	128	86%
Obstetrics	35	23	66%	1184	1065	90%
Infectious diseases	150	52	35%	206	127	62%
Medicine 1^st^	432	125	29%	550	336	61%
Medicine 2nd	237	60	25%	345	214	62%
Medicine 3rd	206	47	23%	291	183	63%
Orthopedics	71	56	79%	278	221	79%
Urology	38	11	29%	281	204	73%
Total	1437	505	35%	4038	3022	75%

**Table 4:** Correlations between albumin, lymphocytes, co-morbidities, and length of hospital stay

	Hospital days	Albumin	Lymphocytes	N° Comorbidities
Hospital days	1	-0.22251	-0.06075	0.31493
		<.0001	0.0221	<.0001
Albumin	-0.22251	1	0.07475	-0.21698
	<.0001		0.0049	<.0001
Lymphocytes	-0.06075	0.07475	1	-0.01401
	0.0221	0.0049		0.5981
N° Comorbidities	0.31493	-0.21698	-0.01401	1
	<.0001	<.0001	0.5981	

Number of patients = 1428

### Stratification of results by hospital ward

The correlation between low levels of albumin and LOS applies to the wards of surgery, infectious diseases, medicine, orthopedics and urology (Table 5); in contrast, the rare measurement of albumin in gynecology and obstetrics unit does not allow to achieve statistical significance. Only in surgery and orthopedics wards, low lymphocyte counts significantly correlated with longer LOS. In all the other wards the relationship between lymphocytes and length of stay is not significant.

**Table 5:** Correlation between albumin and lymphocytes and length of hospital stay, by ward

Ward		Albumin			Lymphocytes	
	Patients	r	*p*	Patients	r	*p*
Surgery 1^st^	101	-0.40901	<0.0001	378	-0.13963	0.0065
Surgery 2^nd^	160	-0.21696	0.0059	326	-0.03280	0.5551
Gynecology		0.08426	0.8575	111	-0.11816	0.2168
Obstetrics		-0.02260	0.8975	743	-0.03041	0.4078
Infectious diseases	150	-0.30501	0.0001	203	-0.13200	0.0605
Medicine 1^st^	432	-0.23764	<0.0001	543	-0.07096	0.0986
Medicine _2_nd	237	-0.22700	0.0004	341	-0.09080	0.0941
Medicine _3_rd	206	-0.17835	0.0103	285	-0.09362	0.1148
Orthopedics	71	-0.24059	0.0433	229	-0.17315	0.0086
Urology		-0.39124	0.0151	216	-0. 04059	0.5530

Small samples are indicated in red bold

### Stratification of results by diagnosis

The correlation between albumin and LOS applied to most of the classes of diagnosis identified (Table 6): malignant tumor, infections, bleeding conditions, fractures, pregnancy/childbirth and miscellaneous conditions (Others). Low levels of albumin were not related to increased length of stay only in benign tumors and inflammatory diseases. In the presence of diagnosis of malignant tumor and pregnancy/childbirth, even though there was a correlation between low levels of albumin and length of stay, there was no statistical significance because of the reduced number of parameters available. In the presence of infection, fractures, and pregnancy/ childbirth, there was a correlation between lymphocyte count and length of stay, with no other relationship for other diagnoses between lymphocyte count and length of stay.

**Table 6:** Correlation between albumin and lymphocytes and length of hospital stay, by diagnosis

Diagnosis		Albumin			Lymphocytes	
	Patients	r	*p*	Patients	r	*p*
Malignant tumors	206	-0.16027	0.0214	343	-0.00565	0.9170
Benign tumors		-0.22459	0.5067	85	-0.15118	0.1672
Inflammatory diseases	95	-0.17584	0.0883	206	-0.12299	0.0782
Infections	397	-0.23001	<0.0001	649	-0.12547	0.0014
Hemorrhagic conditions	60	-0.35899	0.0049	131	-0.05823	0.5089
Fractures	71	-0.31096	0.0083	202	-0.14823	0.0353
Pregnancy/Childbirth		-0.52093	0.0064	613	-0.08906	0.0275
Others	567	-0.23845	<0.0001	1146	-0.04780	0.1058

Small samples are indicated in red bold

### Multivariate Analysis and Regression

**Fig. 3:**
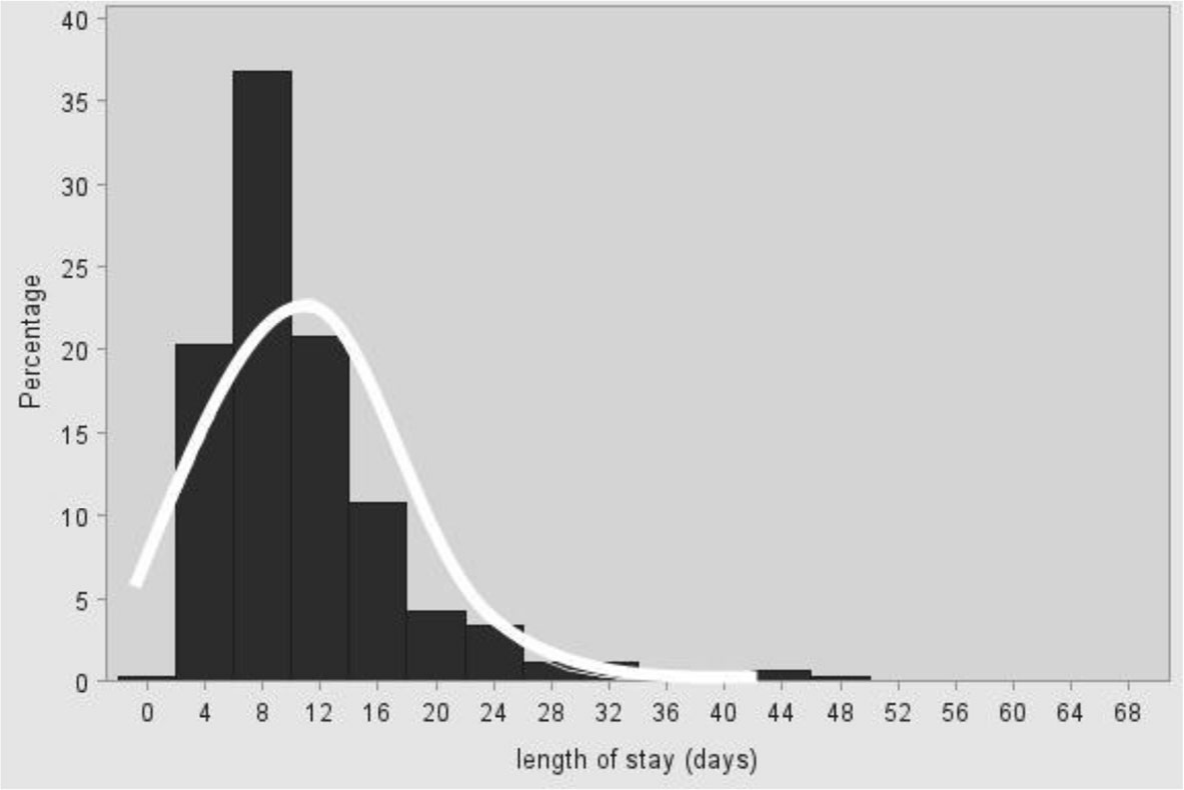
Percentage of patients with a given length of stay (in days). Legend. The *line* describes the fitted Normal distribution

**Fig. 4:**
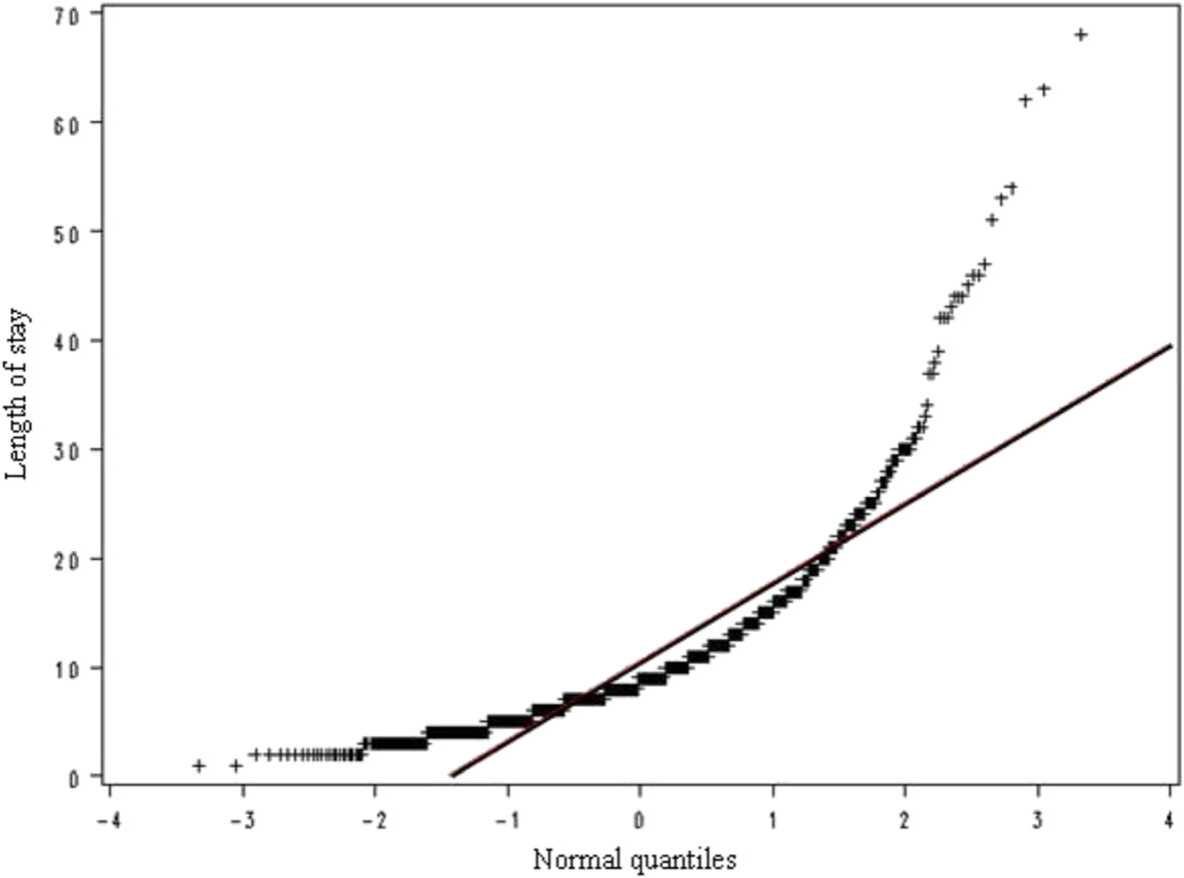
Normal Quantile-Quantile Plot for length of stay. Legend. The plot compares the ordered values of *length of stay (days)* with quantiles of the normal distribution. If the data distribution matches the theoretical distribution, the points on the plot form a linear pattern

From the preliminary descriptive study, we observed that the classic model of linear regression was inadequate for our study due to the distribution of the variable “hospital days” (Figs. 3 and 4). In fact, the classical model requires the assumption that the distribution of the errors (as well as the variables considered) is Normal, condition that does not occur in our case. Therefore we adopted the method ‘stepwise backward’ to better fit our model and our needs to study the effects of each variable involved. In Table 7 we show the parameters studied, according to the method of maximum likelihood. The hypothesis tested in this case is the significance of the variable Albumin and length of hospital stay. Because of the large number of variables involved, multivariate statistical method was required. Factorial analysis through Principal Component method, with a limited loss of information, has eliminated the multi-collinearity between the original variables. Reducing the number of factors considered, regression has shown that platelets, advanced age, number of co-morbidities, diagnosis, gender and type of admission are all significant variables in explaining LOS. In particular, for every unit increase in the number of platelets there was a small increase in length of stay, and the value found for the type of admission indicates that LOS decreased by approximately 19% if recovery changed from “urgent” to “elective”.

**Table 7:** Regression analysis – estimates of maximum likelihood

Parameter	Level_1	Estimate	Standard Error	Lim. Conf. < 95%	Lim. Conf. > 95%	*x*^2^ Wald	Pr > *x*^2^
Albumin		-0.1479	0.0473	-0.2405	-0.0553	9.79	0.0018
Lymphocytes		0.0717	0.0415	-0.0097	0.1530	2.98	0.0843
Number of Comorbidities		0.2625	0.0255	0.2125	0.3126	105.76	<0.0001
Red Blood Cells		0.1008	0.0428	0.0170	0.1846	5.56	0.0184
Platelets		0.0008	0.0003	0.0001	0.0014	5.88	0.0154
Diagnosis 1	Infections	-0.0334	0.1631	-0.3531	0.2863	0.04	0.8376
Diagnosis 2	Malignant tumors	0.0600	0.1208	-0.1768	0.2967	0.25	0.6195
Diagnosis 3	Benign tumors	0.4561	0.2490	-0.0320	0.9441	3.35	0.0670
Diagnosis 4	Other	-0.0107	0.1310	-0.2675	0.2461	0.01	0.9347
Diagnosis 5	Fracture	0.2357	0.1305	-0.0200	0.4913	3.26	0.0708
Diagnosis 6	Pregnancy and childbirth	-0.5629	0.2357	-1.0249	-0.1009	5.70	0.0169
Diagnosis 7	Haemorrhagic conditions	-0.0037	0.1764	-0.3494	0.3420	0.00	0.9834
Diagnosis 8	Inflammatory diseases	0	0	0	0	.	.

For the variable “Diagnosis” the probability of incurring in a longer length of stay is higher in case of fractures; the risk increases by 24% passing from the diagnosis of malignant tumor to fracture, but in the case of inflammatory diseases the coefficient appears not significant. We can conclude that all variables shown in Table 8 are highly significant in explaining patients’ length of stay. Finally, also patients gender helps to explain the phenomenon analyzed; under this aspect, women had a lower risk of incurring in a long hospital stay, as it is not taken into account in the regression model.

**Table 8:** Likelihood ratio – significance analysis

Origin	_*X*_ *2*	Pr > *X* ^*2*^	Index	Hospital days
Platelets	29.14	<0.0001	↑	↑
Age	52.23	<0.0001	↑	↑
Number of Co-morbidities	289.12	<0.0001	↑	↑
Diagnosis	125.36	<0.0001	Inflammatory diseases excluded	
Gender	8.33	0.0039	Male	↑
Recovery	21.15	<0.0001	Urgent	↑

About the Discriminant function analysis, which compared patients with and without albumin, we found that only 7 of the 16 variables initially selected as discriminant inputs are effectively correlated with the measurement of the albumin parameter (Table 9). Therefore, we can state that in the group with albumin value, patients have higer age at admission, increased hospital stay (number of days), a higher concentration of platelets (more than 450 10^3/uL) and eosinophils (higer than 0.5 10^3/ul), but lower level of White Blood Cells (less than 4.0 x 10^3/ul) and MCHC (lower than 31.5 g/dl). Furthermore, we can’t say with sufficient certainty whether the type of surgery have significantly influenced patients’ length of stay. In the sample studied, the surgical procedures performed were 534 and highly heterogeneous (251 different types of surgical interventions). This obstacle made it difficult to group or analyze the procedures in relation to LOS, so we are not able to draw any conclusions of scientific or statistical value.

**Table 9:** Stepwise selection for patients with albumin value

Summary of stepwise selection								
Step	Input	R^2^ (partial)	F Value	Pr > F	Lambda Wilks	Pr < Lambda	Average Squared Canonical Correlation	Pr > ASCC
1	Age at recovery	0,1664	658,77	<,0001	0,8336	<,0001	0,1664	<,0001
2	Days of recovery	0,0454	156,69	<,0001	0,7957	<,0001	0,2043	<,0001
3	White blood count (WBC) 10^3/ul 4.0 – 10.0	0,0049	16,31	<,0001	0,7918	<,0001	0,2082	<,0001
4	Monocytes (absolute nr) 10^3/ul 0.2 – 1.0	0,0024	8,03	0,005	0,7899	<,0001	0,2101	<,0001
5	Mean corpuscular hemoglobin concentration (MCHC) g/dl 31.5 – 34.5	0,0021	6,84	0,009	0,7883	<,0001	0,2117	<,0001
6	Platelets (PLT) 10^3/uL 150 – 450	0,0017	5,58	0,018	0,7869	<,0001	0,2131	<,0001
7	Eosinophils (absolute nr) 10^3/ul 0.0 – 0.5	0,0012	3,81	0,051	0,7860	<,0001	0,2140	<,0001

## Discussion

### Summary: aims and main results

The correlation between reduced albumin levels and prolonged hospitalization applied to the entire sample and to almost all hospital wards (except for gynecology and obstetrics), and also to certain diseases like benign tumors and inflammatory diseases. Even though a low blood lymphocyte count was associated with longer LOS in the entire sample, the results were only significant in the ward of orthopedics and surgery. Only for the diagnosis of certain clinical conditions like infectious diseases, fractures and pregnancy/childbirth, a low blood lymphocyte count correlated with prolonged hospital stay. Using the regression method platelets, advanced age, number of co-morbidities, diagnosis, gender and type of admission were significant variables associated with prolonged hospital stay, yielding important information that could be used to improve current nutritional screening tools.

### Possible mechanisms and explanations for the findings

Our results indicate that low albumin levels have a close correlation with longer hospital stays, while the relationship with lymphocyte count is only marginal, in accordance with previous studies [17]. Low lymphocyte counts lead to prolonged hospitalization mainly in the presence of infectious diseases, probably because lymphocytes play a critical role in the immune response against the pathogens involved. In the orthopedics ward, a decreased lymphocyte count leads to a significant increase in hospital days, mainly due to trauma and/or inflamation [7, 18, 19]. ACTH, corticosteroids, catecholamines, cytokines, chemokines and allarmines play an important role in the initiation and maintenance of the inflammatory response to injury, and also in the regulation of the albumin gene expression [8]. The complex network of cytokines appears to be disrupted, especially after surgery [20]. Interleukin-1β, -6, -8 and TNF-α, some suppressive cytokines such as Interleukin-10, -4 and Interleukin-1 receptor antagonist significantly increase just after the beginning of surgery, while Interferon-γ and Interleukin-2 are markedly reduced [20]. This imbalance alters the immune response and may in part explain the correlation between low levels of lymphocytes and length of stay found in surgery ward I.

### Strengths and limitations of the study

The main strengths of this study consist in the large amount of data at our disposal, and in the homogeneity of the sample, obtained through the standardization linked to the use of patient electronic charts; this allowed to identify various pathologies and co-morbidities through the use of international codes. The results obtained confirm the hypothesis that low albumin levels and low lymphocyte counts are associated with increased length of stay; the relationship applied to the entire sample examined, and the strength of correlation was higher in some wards, and might be applied to any kind of hospital.

This study has also highlighted some critical points of the clinical approach: limited evaluation of albumin at admission, and inability to trace the anthropometric data of patients. Anthropometric data and albumin levels were regularly measured before surgical procedures, in order to properly administer general anesthesia, antibiotics, or chemotherapeutic drugs, but not to determine the patient general health status. Clinicians carelessness about nutritional status has led to a general disinterest for important parameters such as weight, height, BMI and selected biochemical markers [21, 22]. Albumin measurement is an important low-cost instrumental exam, still marginally used in hospitals. Also anthropometric data are important for the assessment of nutritional status, but they are rarely measured, even though recent studies confirmed that these parameters represent the most predictive factor in determining the risk of complications: for instance, BMI values below 20 kg/m^2^ determine hospital stays 2.1 times longer compared to hospitalization for BMI within normal range; albumin and lymphocytes severely affect the length of hospitalization [23–26].

### The importance of an early assessment of albumin and lymphocytes

The present study has highlighted the importance of an early assessment of albumin levsls. Low levels of albumin can be caused by various factors, like decreased food and calorie intake due to hospitalization [27], prolonged fasting [27], surgical operations [19], postural changes [13], cytokines [13], drugs [28], hormonal therapies [19], inflammatory diseases, liver and kidney diseases, cancer, infections and all medical conditions characterized by high metabolic energy requirement. Sepsis, for instance, can influence synthesis, consumption, and distribution of albumin between intra and extravascular compartments [11, 12, 18]. Rapid changes in albumin levels are common after admission, and the “sink rate” is very rapid especially after admission. Researchers have suggested that the sink rate is too fast to be associated only with patients nutritional deterioration [26]. Posture modification, from standing to reclined position, was shown to cause a decrease in serum albumin: the production of TNF-α, Interleukin-2 and -6, inhibits the synthesis of albumin by acting on its gene expression, through a down-regulation process [13]. Since lymphocytes are involved in the immune response, decreased lymphocyte counts are considered an indicator of the general decline of physiological functions, and factors of poor prognosis in hospitalized patients [11].

### Clinical implications and future perspectives

With this research we intended to raise clinicians attention to the importance of albumin and lymphocytes to assess the status of hospitalized patients. It would have been useful and important to evaluate also patient anthropometric parameters which would have given the study a more complete analysis. These health indices, once routinely recorded by nursing staff, are now considered as the major reason of wasting time dedicated to more invasive clinical procedures. Therefore in light of the fact of there being no screening method universally accepted, we aim to provide guidelines for the assessment of patients health status in different contexts (community, hospitals, patients with specific diseases, etc). Low albumin levels and low lymphocytes count are directly related to longer hospital stays, and therefore the alteration of these markers causes a further deterioration of the nutritional status, thus promoting a cycle of malnutrition-infection leading to the onset of protein-energy malnutrition, a self-perpetuating process with a strong influence on patient outcomes (poor wound healing, loss of muscle strength, decreased mobility, increased risk of thromboembolic complications [27, 28]), length of stay and associated costs [29, 30].

Our challenge for the future is to convince clinicians on the importance of assessing albumin and lymphocytes and eventually doing a whole nutritional assessment of the single patient.

## Conclusion

We can conclude portraying the data of a study conducted in 2003 in which it has been described that with a reduction of 1 g/dl in serum albumin, hospital days had increased by 71% [31]. This finding grabbed our attention; in our study 924 patients had an albumin concentration below the lower limit (3.5 g/dl) of which 750 with a reduction of 1 g/dl, 169 with a decrease of 2 g/dl and 5 with less than 3 g/dl. Considering that for each gram lost of albumin there is a theoretical probability 71% increase in longer hospitalization and that albumin in our sample was evaluated in only 36% of our cases, and assuming that the distribution of the decline of albumin may be similar even in the population which albumin has not been evaluated, there are three questions to be asked: how many days patients have unnecessarily spent in hospital? How much have these additional days of hospitalization weighed, from an economic point of view, in the absence of albumin assessment? And more important, how much the extra days of hospitalization would have not economically impacted the hospital with an appropriate nutritional assessment? Exploiting new clinical knowledge and investing time to ensure maximum therapeutic benefit for each patient means to act in the interest of the community. One should invest time and resources during the first steps of admission, in order to assess patients real health status, and to create a personalized plan for recovery. This important concept is the rationale that guided us to conduct this research, but it should also be the reason that accompanies any decision of each health care professional in his profession.

## Abbreviations

BIA: Body impedance assessment
GLM: Generalized linear models
LOS: Length of stay
