# Correction to: Length of hospitalization is associated with selected biomarkers (albumin and lymphocytes) and with co-morbidities: study on 4000 patients

**DOI:** 10.1186/s40364-020-00255-8

**Published:** 2021-01-08

**Authors:** Antonio E. Pontiroli, Lara Loreggian, Marco P. L. Rovati, Elena De Patto, Laura Folini, Federico Raveglia, Matilde De Simone, Alessandro Baisi, Ugo Cioffi

**Affiliations:** 1 2nd Division of Medicine, Ospedale San Paolo and University of Milan, Milan, Italy; 2 2nd Division of Surgery, Ospedale San Paolo and University of Milan, Milan, Italy; 3 Division of Thoracic Surgery, Ospedale San Paolo and University of Milan, Milan, Italy; 4 Department of Surgery, University of Milan, Milan, Italy

**Correction to: Biomark Res (2017) 5:13**

**https://doi.org/10.1186/s40364-017-0091-x**

The datasets used and/or analysed during the current study are no longer available as supplemental material and are now available from the corresponding author on reasonable request [1].
